# Effects of hypertonic saline on macrophage migration inhibitory factor in traumatic conditions

**DOI:** 10.3892/etm.2012.800

**Published:** 2012-06-11

**Authors:** JUNG-YOUN KIM, SUNG-HYUK CHOI, YOUNG-HOON YOON, SUNG-WOO MOON, YOUNG-DUCK CHO

**Affiliations:** Department of Emergency Medicine, College of Medicine, Korea University, Seoul 152-703, Republic of Korea

**Keywords:** macrophage migration inhibitory factor, macrophage, neutrophil, trauma

## Abstract

Trauma-induced suppression of cellular immune function contributes to sepsis, multiple organ dysfunction syndrome (MODS) and mortality. Macrophage migration inhibitory factor (MIF) has been revealed to be central to several immune responses. However, the role of MIF in trauma-like conditions is unknown. Therefore, the present study evaluated MIF in macrophages and polymorphonuclear neutrophils (PMNs). The effects of hypertonic saline (HTS) on lipopolysaccharide (LPS)-induced MIF levels were evaluated in macrophages. MIF concentrations were determined by an enzyme-linked immnosorbent assay (ELISA) and cell lysates were used for western blot analysis. The effects of HTS on *N*-formyl-methionyl-leucyl-phenylalanine (fMLP)-induced MIF were evaluated in PMNs. MIF concentrations were determined by ELISA, western blotting and real time-polymerase chain reaction (RT-PCR) to determine MIF expression. MIF levels, which were measured by the ELISA, increased by 1.24±0.38 ng/ml in the supernatants of LPS-stimulated macrophages compared with the controls (0.79±0.07 ng/ml) at 2 h. HTS10 (150 mmol/l) partially restored MIF levels (0.84±0.22 ng/ml; P<0.05). Also, western blotting was performed and MIF protein levels were higher in the LPS-stimulated macrphages (20% increase in band density) compared with the controls (P<0.05). The addition of HTS decreased MIF protein expression. MIF levels in fMLP-stimulated PMN cells were unchanged compared with the controls according to the ELISA, western blotting and RT-PCR. No effects were observed following treatment with HTS. MIF concentrations and MIF expression were higher in LPS-stimulated macrophages than controls and HTS restored MIF levels to those of the controls. MIF levels were unchanged in PMNs stimulated by fMLP.

## Introduction

Trauma-induced suppression of the cellular immune function likely contributes to sepsis, multiple organ dysfunction syndrome (MODS) and mortality. Hypertonic saline (HTS) is known to have anti-inflammatory effects. After substantial of blood loss, trauma patients often experience severe post-traumatic complications, such as acute respiratory stress syndrome, multiple organ failure and sepsis (1,2). HTS resuscitation decreases the probability of sepsis following hemorrhagic shock (3,4) and studies have shown that HTS is a simple but effective tool for modulating immune function following trauma (5–8). Ischemia and reperfusion primes neutrophils and mononuclear cells to produce an excessive response to inflammatory stimuli in post-traumatic patients (the ‘two-hit’ hypothesis) (9). Prevention of exaggerated inflammation and immunosuppression has been a topic of trauma research for a number of years. HTS has attracted attention as a possible therapeutic approach for managing harmful immune responses in trauma patients, particularly those associated with neutrophil function (10–14). Macrophage migration inhibitory factor (MIF) has been revealed to be central to several immune responses, including the modulation of numerous cytokines and monocyte, neutrophil and T cell activation. MIF may be a general marker for systemic inflammation in septic acute critical illness. By controlling immune and inflammatory responses, MIF is considered to be important in the pathophysiology of septic shock and chronic inflammatory diseases (15). However, the role of MIF in trauma-like conditions is unknown. Therefore, the present study was conducted to evaluate MIF in macrophages or polymorphonuclear neutrophils (PMNs), in response to early phase injury following stimulation with lipopolysaccharide (LPS) to induce infection conditions or *N*-formyl-methionyl-leucyl-phenylalanine (fMLP) to induce trauma-like conditions, either in the presence or absence of HTS.

## Materials and methods

### THP-1 cells

#### Culture and treatment of cells

THP-1 cells (American Type Culture Collection TIB-202, Manassas, VA, USA), an immortalized human monocytic cell line, were differentiated to macrophages as previously described with a few modifications (16). Cells were treated with 162 μmol/ml phorbol 12-myristate 13-acetate (PMA; Sigma-Aldrich Co., St. Louis, MO, USA) for 72 h at 37°C, 5% CO_2_. Differentiated cells were washed three times with HBSS (Gibco, Carlsbad, CA, USA), removed from the plate with 0.25% trypsin-EDTA (Gibco) and seeded in 96-well plates for an enzyme-linked immunosorbent assay (ELISA) and 24-well tissue culture plates for western blotting at 2x10^6^/ml viable cells per well in complete media. THP-1 monocyte-derived macrophages were treated with LPS at different tonicities. The effect of HTS on LPS-induced MIF was evaluated in macrophages with 1 μg/ml LPS. HTS at 10, 20 or 40 mmol/l above isotonicity (140 mmol/l) was added. This study was approved by the Institutional Review Board from Korea University Guro Hospital (NO.KUGH – 10157).

#### Enzyme-linked immunosorbent assay for MIF

Supernatants were collected after incubation for 2 or 20 h. The MIF concentration in the culture supernatants was measured by sandwich ELISA. Briefly, 2 μg/ml of monoclonal capture antibody (R&D Systems, Minneapolis, MN, USA) was added to a 96-well plate and incubated for one day at room temperature and washed with buffer three times. After washing, the plates were incubated in a blocking solution of phosphate-buffered saline (PBS) containing 1% bovine serum albumin (BSA) and 0.05% Tween-20 for 1 h at room temperature and washed with buffer three times. Test samples and standard recombinant MIF (R&D Systems) were added to the plates and incubated for 2 h at 4°C. Plates were washed three times with PBS containing Tween-20, 200 ng/ml of biotinylated detection monoclonal goat-antihuman antibodies (R&D Systems) were added and the plates were incubated for 2 h at room temperature. After washing three times, streptavidin-alkaline-phosphatase (1:2000; Sigma-Aldrich Co.) was added and the reaction was allowed to proceed for 20 min at room temperature. The plates were washed three times and 1 mg/ml of p-nitrophenylphosphate dissolved in diethanolamine (Sigma-Aldrich Co.) was added to induce a color reaction which was stopped with 50 μl of 1 M NaOH. The optical density at 450 nm was measured on an automated microplate reader (Bio-Rad Laboratories Inc., Hercules, CA, USA). A standard curve was generated by plotting the optical density vs. the log of the MIF concentration. Experiments were conducted 10 times.

#### Protein extracts and western blot analysis for MIF expression

Following incubation for 24 h at 37°C, cells were washed twice in cold PBS and centrifuged for 10 min. Cell pellets were resuspended in 10 μl per 2×10^6^ cell/ml of superlysis buffer [protease inhibitors, 1 M 4-(2-hydroxyethyl)-1-piper-azineethanesulfonic acid (HEPES), 5 M NaCl, 0.5 M ethylenediaminetetraacetic acid (EDTA), 1 mM Na_3_VO_4_, 20% Triton X-100, 50 mM phenylmethylsulfonylfluoride], incubated on ice for 7 min and centrifuged at 3,000 × g for 15 min at 4°C. The total protein concentration was determined by the Bradford method using a commercially available assay kit (Thermo Fisher Scientific, Rockford, IL, USA) (8). Prepared protein lysates were aliquoted and used for western blot analysis. Proteins (5 μg/ml) were fractionated on a 15% sodium dodecylsulfate-polyacrylamide gel (Bio-Rad Laboratories Inc.) and transferred to a nitrocellulose membrane. Membranes were blocked for 1 h in 5% BSA (Sigma-Aldrich Co.) and incubated with anti-human MIF (1:250; R&D systems). After washing, membranes were incubated with 1:2,000 horseradish peroxidase-labeled goat anti-mouse antibody (R&D systems). Proteins were detected using a SuperSignal (Thermo Fisher Scientific Inc.) chemiluminescence kit.

### PMN cells

#### Separation and stimulation of PMNs

PMNs were separated using a modified Boyum method. After obtaining consent, venous blood samples were collected directly into a tube containing preserved EDTA from 10 healthy volunteers. From each collected whole blood sample, 5 ml was aliquoted into 15 ml test tubes with 5 ml of Polymorphprep (Nycomed Pharma AS, Oslo, Norway), followed by centrifugation for 37 min at 500 x g. The PMN cell layer between the monocyte and red blood cell layers was collected. To remove the remaining red blood cells, samples were incubated with 0.2% saline solution for 30 sec, after which a 1.8% saline solution was added to create 0.9% normal osmotic pressure. Samples were centrifuged at 450 × g for 10 min, followed by two washes with PBS. Separated PMNs were incubated in RPMI-1640 medium containing penicillin and supplemented with fetal bovine serum (FBS; 10%) and HEPES. A final concentration of 1×10^6^ cell/ml with viability >95% was demonstrated using trypan blue dye. fMLP (43.76 mg; Sigma-Aldrich Co.) was dissolved in dimethyl sulfoxide (10 ml; DMSO, Sigma-Aldrich Co.) to 1 μM. PMNs were stimulated using fMLP. The prepared neutrophils were divided into 5 groups. The control group received no stimulation and isotonic conditions (140 mmol/l) were maintained. Another group was stimulated with fMLP but maintained at isotonic conditions. Three groups had hypertonic conditions of 10, 20 and 40 mmol/l above isotonicity with HTS added following stimulation with fMLP. MIF concentrations in the supernatant were determined by the ELISA, while cell lysates were used for western blotting and real time-polymerase chain reaction (RT-PCR) to determine MIF expression. The ELISA and western blotting for MIF were conducted as described above.

#### RT-PCR

Expression of MIF mRNA was detected by quantitative (q)RT-PCR. Total RNA was extracted from cells using TRIzol reagent according to the manufacturer's instructions (RNeasy Mini Kit, Qiagen, Hilden, Germany) and the concentration of the sample in diethypirocarbonate-treated water was determined. Extracted RNA was stored at −70°C and treated with DNase I prior to use. qRT-PCR was employed to detect MIF mRNA. The total RNA (200 ng) from each sample was reverse transcribed into complementary DNA (cDNA) using a high-capacity cDNA reverse transcription kit. The final reaction volume was 10 μl. After completion of the first-strand cDNA synthesis, the MIF probe was used to analyze 34 cycles of 50°C for 2 min, 95°C for 10 min, 95°C 15 min and 60°C for 1 min, with an RT-PCR system (AB7300, Applied Biosystems, Foster City, CA, USA).

#### Data and statistical analysis

Measurements are presented as mean with standard deviation. The Student's t-test and one-way ANOVA were used for the statistical analysis. P<0.05 was considered to indicate statistically significant differences.

## Results

### 

#### Effect of HTS on MIF concentration in LPS-induced macrophage supernatants

**Figure 1:**
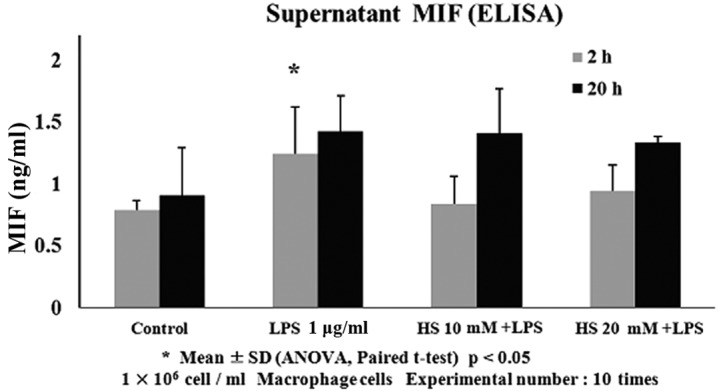
MIF concentration in macrophage cell supernatants by ELISA. Macrophages in the presence of LPS had increased MIF level (1.24±0.38 ng/ml) compared with macrophages alone (0.79±0.07 ng/ml). HTS10 decreased MIF levels (0.83±0.22 ng/ml) compared with LPS-stimulated macrophages (P<0.05). LPS-stimulated macrophages treated with HTS20 had lower MIF levels (0.94±0.21 ng/ml). However, MIF levels between HTS10- and HTS20-treated groups were not significantly different. Experiments were conducted 10 times. 1x10^6^ cell/ml, one-day incubation. MIF, migration inhibitory factor; ELISA, enzyme-linked immnosorbent assay; LPS, lipopolysaccharide; HTS, hypertonic saline.

To understand the association between HTS and MIF in LPS-induced macrophage cells, the MIF levels were measured in cell supernatants (Fig. 1). The MIF levels increased by 1.24±0.38 ng/ml in the supernatant of LPS-stimulated cells compared with the control level of 0.79±0.07 ng/ml at 2 h incubation. HTS decreased the MIF levels to 0.84±0.22 ng/ml in LPS-stimulated macrophage cells at 10 mM above hypertonicity (P<0.05). HTS decreased the MIF levels to 0.94±0.21 ng/ml in LPS-stimulated macrophage cells at 20 mM above hypertonicity (P<0.05). MIF levels between the HTS10- and HTS20-treated cells were not significantly different. The groups incubated for 20 h were not significantly different from groups incubated for 2 h. Although the MIF level of the 20-h stimulated group increased to 1.42 ng/ml ± 0.29, compared to the control group with 0.91 ng/ml ± 0.38, this was not a statistically significant. In addition, there was no statistical difference between 1.42 ng/ml ± 0.35 at 10 mM above isotonicity and 1.33 ng/ml ± 0.05 at 20 mM above isotonicity with added HTS.

#### Effect of HTS on MIF expression in LPS-induced macrophages

**Figure 2:**
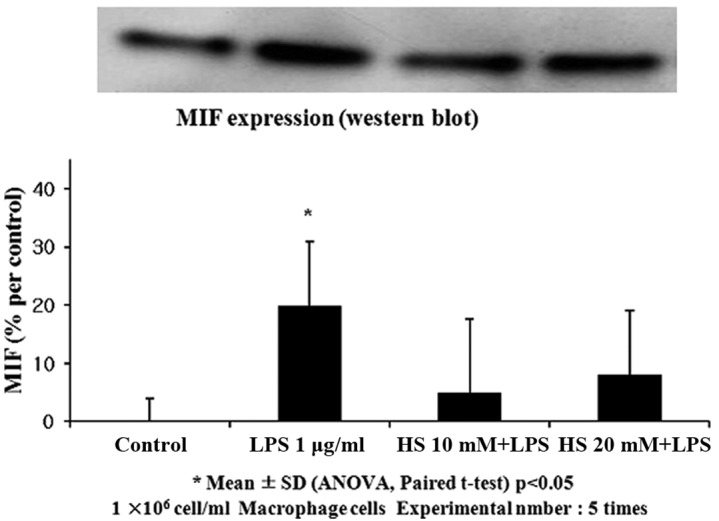
MIF expression in macrophages by western blotting. Levels of MIF protein were higher in LPS-stimulated cells (20% increase in band density; P<0.05). The addition of HTS to LPS-stimulated cells decreased MIF protein with the lowest expression in the HTS10-treated cells. 1x10^6^ cell/ml, one-day incubation, MIF MW: 12.5 kDa. MIF, migration inhibitory factor; LPS, lipopolysaccharide; HTS, hypertonic saline.

To determine the effect of HTS on MIF expression, western blotting was performed (Fig. 2). Correlating with the ELISA results, the levels of MIF protein were higher in the LPS-stimulated cells (20% increase in band density; P<0.05). The addition of HTS to the LPS-stimulated cells decreased the MIF protein at 10 mM and 20 mM above isotonicity, However, MIF expession between the HTS10- and HTS20-treated cells was not significantly different.

#### Effect of HTS on MIF concentration and MIF expression in PMN cells with fMLP stimulation

**Figure 3:**
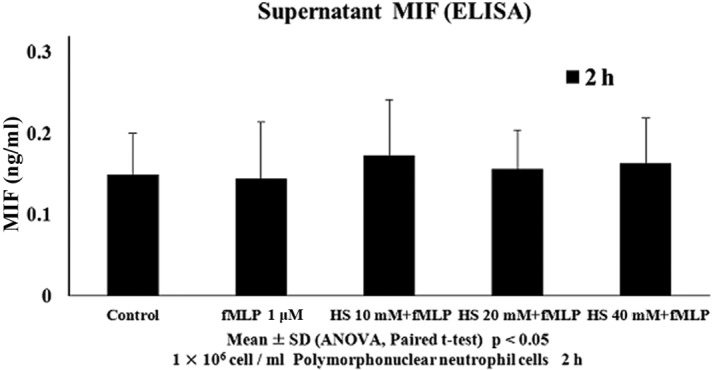
MIF concentration in PMN cell supernatants by ELISA. MIF levels were measured in the PMN cell supernatants. MIF levels in fMLP-stimulated cells were not changed compared with controls. MIF levels in the HTS10- and HTS20-treated groups were not significantly different. Experiments were conducted 10 times. 1x10^6^ cell/ml, one-day incubation. MIF, migration inhibitory factor; PMN, polymorphonuclear neutrophil, ELISA, enzyme-linked immnosorbent assay; fMLP, *N*-formyl-methionyl-leucyl-phenylalanine; HTS, hypertonic saline.

**Figure 4:**
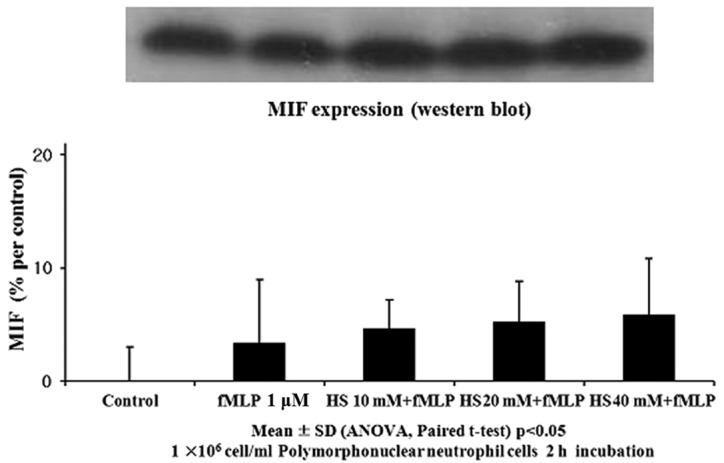
MIF expression in PMNs by western blotting. MIF protein was measured in PMNs. MIF levels in fMLP-stimulated cells were unchanged compared with controls. MIF levels in HTS10- and HTS20-treated groups were not significantly different. Experiments were conducted 10 times. 1x10^6^ cell/ml, one-day, incubation. MIF, migration inhibitory factor; PMN, polymorphonuclear neutrophil; fMLP, *N*-formyl-methionyl-leucyl-phenylalanine; HTS, hypertonic saline.

**Figure 5:**
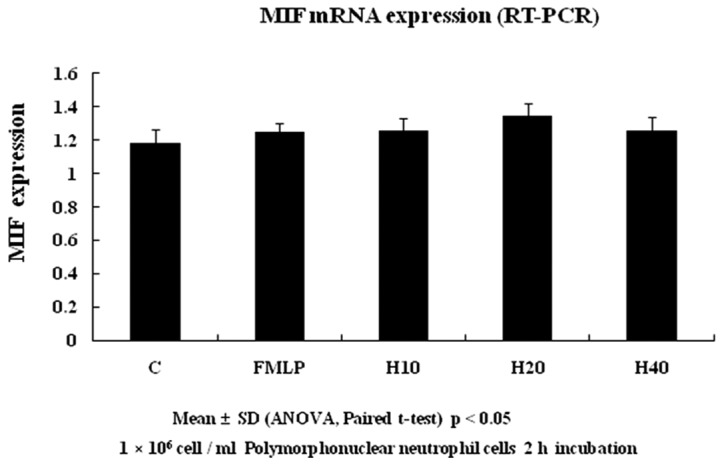
MIF in PMNs by RT-PCR. MIF mRNA was measured in PMNs. MIF levels in fMLP-stimulated cells were unchanged compared with controls. MIF levels in HTS10- and HTS20-treated groups were not significantly different. Experiments were conducted 10 times. 1×10^6^ cell/ml, one-day, incubation. MIF, migration inhibitory factor; PMN, polymorphonuclear neutrophil; fMLP, *N*-formyl-methionyl-leucyl-phenylalanine; RT-PCR, real-time polymerase chain reaction; HTS, hypertonic saline.

PMN cells were plated on 96-well culture plates at a concentration of 1x10^6^ cell/ml in cell culture media (Figs. 3–5). To determine the association between HTS and MIF in fMLP-induced PMN cells, the MIF levels were measured in cell supernatants. The MIF levels in fMLP-stimulated cells were unchanged compared with the controls. In correlation with the ELISA results, the levels of MIF expression were unchanged in the western blotting and RT-PCR. Treatment with HTS had no effect.

## Discussion

The present study demonstrated that MIF increased in macrophages stimulated with LPS and decreased with HTS treatment. It also demonstrated a lack of increase in MIF levels in neutrophils stimulated with fMLP, unlike the MIF reaction in macrophages stimulated with LPS. Few studies have examined the association between the expression of MIF and HTS in cells.

A leading cause of late mortality in trauma patients is MODS resulting from the deregulation of the immune-inflammatory response and homeostasis. Following major trauma, a large number of pro- and anti-inflammatory cytokines are released by activated monocytes/macrophages and PMNs. In a healthy patient with a minor injury, homeostasis is restored quickly and the inflammatory response remains incomplete. In the case of a major trauma, however, the inflammatory response may proliferate throughout the whole body, leading to multiple organ injury, including acute respiratory distress syndrome (ARDS), sepsis, septic shock and MODS with a concomitant significant morbidity and mortality for the injured patient (2,17).

Hemorrhagic shock is a leading cause of early mortality in trauma patients. Resuscitation from traumatic blood loss activates the innate immune system, potentially leading to ARDS and MODS. HTS is a safe and efficient fluid for resuscitation from hemorrhagic shock and reducing the intracranial pressure in patients with brain injuries (18), with a number of potentially beneficial immunomodulating effects (8,19). HTS (7.5% saline) has been shown to modulate the entire systematic immunoinflammatory response following injury (20), as well as decrease cytokine production by monocytes and blunt neutrophil activation induced by shock (21–24). The decrease in cytokine release may be an additional beneficial effect of HTS on the immune function in the resuscitation of post-traumatic patients (25).

Cytokines are important in modulating the host immunoinflammatory responses to infection and trauma. They regulate the first nonspecific phase of the host response by combining a local inflammatory reaction and control the next specific immune response. In 1966, historical experiments by Bloom and Bennett (26) and David (27) first identified MIF as a nondialyzable protein factor produced by sensitized lymphocytes that was associated with delayed-type hypersensitivity. MIF was characterized by the ability of crude extracts to inhibit the random migration of guinea pig peritoneal exudate macrophages *in vitro*(26,27) and subsequently activate macrophage function (28,29). In spite of these observations describing cytokine activity >30 years ago, a detailed view of the biological function of MIF remained elusive until the classical T cell cytokine macrophage MIF reemerged as a critical mediator of the host immune and stress response (15).

LPS is the major component of the outer membrane of Gram-negative bacteria, contributing to the structural integrity of the bacteria and protecting the membrane from certain types of chemical attack. LPS is important as mutation or removal results in the death of Gram-negative bacteria. LPS is an endotoxin and induces a strong response from normal animal immune systems. It has also been implicated in nonpathogenic aspects of bacterial ecology, including surface adhesion, bacteriophage sensitivity and interactions with predators such as amoebae. LPS acts as a prototypical endotoxin since it binds the CD14/TLR4/MD2 receptor complex, which promotes the secretion of proinflammatory cytokines in numerous cell types, particularly macrophages and B cells. In immunology, the term ‘LPS challenge’ refers to the process of exposing a subject to an LPS that may act as a toxin. LPS is also an exogenous pyrogen (external fever-inducing substance) (30,31).

As an inflammation-triggering agent, the fMLP used to stimulate PMNs cells in the present study is a synthetic chemoattractant. It is a formyl peptide secreted in an area of inflammation during bacterial infection that binds to its receptor on the surface of neutrophils, stimulating cytokine secretion. Thus, fMLP is generally accepted as inducing a reaction similar to the inflammatory response (32).

In the results of the present study, MIF expression was demonstrated to be increased in macrophages stimulated by LPS and decreased by HTS. According to Choi *et al*(33), MIF expression changes in T cells treated with HTS and this is associated with T cell dysfunction. This is consistent with the results for MIF in macrophages in the present study. However, no significant changes of MIF in PMN cells were observed, regardless of stimulation or HTS. In confirming the level of TNF-α by stimulating PMN cells with fMLP and LPS, Vulcano *et al*(30), detected no secretion when PMN cells were stimulated with fMLP, but secretion when cells were stimulated with LPS. Vulcano *et al*(30) proposed that the difference was due to dynamic processes at the LPS and fMLP binding sites. Studies have described the independent actions of LPS and fMLP or a priming effect exerted by LPS on a different agonist. However, in general, the sequence of interaction between these two bacterial components in the regulation of inflammation is not fully undersood (30). An increase in MIF was observed in macrophages stimulated by LPS. Similar conditions were observed in a study by Schmidt-Supprian *et al*(34), with processing with activated protein C afterwards. The authors reported that activated protein C reduces MIF levels, similar to HTS.

In the present study, RT-PCR data for macrophages stimulated with LPS were not acquired, since MIF exhibited a significant difference by ELISA and western blotting. RT-PCR was used in the PMN-fMLP experimental group, since no significant difference was observed by ELISA or western blotting.

As in the majority of studies that focus on trauma and injury conditions, the present study was conducted by stimulating PMN cells with fMLP and LPS was mainly used to study infection conditions, respectively. Therefore, each experimental group was considered to represent trauma and infection conditions. The results of the present study suggested that MIF is not expressed under simple trauma conditions but is expressed in conjunction with infection. In this case, MIF should be considered to be an important cytokine in the dividing stages and estimating prognosis by the increase in infection rate, progression to sepsis and MODS and mortality in cases of immune deficiency caused by trauma. If the differences in the results reflect the differences between infection, represented by macrophages and LPS, and trauma, represented by PMNs and fMLP, no MIF expression is expected in cases of trauma and only MIF would be expressed in cases where trauma is combined with infection.

The limitations of the present study were, firstly, the *in vitro* design may be different from complex situations *in vivo* with various cytokines and a diverse environment. Secondly, although the difference between the results of PMN cells and macrophages was interpreted to be caused by differences in stimulants, it may be attributable to a difference in the cells themselves. As both cells and stimulants differed, a comparison between the two results is expected to be insignificant. However, since almost nothing is known concerning the expression of MIF in trauma, infection, inflammation or other conditions, the importance of the present study is in the results about the expression of MIF caused by stimulation in macrophages and the findings of a reaction with HTS and no expression of MIF in PMNs.

The present study demonstrated that MIF increased in LPS-stimulated macrophages and decreased with HTS treatment. However, it also demonstrated that the level of MIF was not associated with stimulation or HTS for PMN cells stimulated with fMLP. Inflammation and immune modulation by HTS occurs, at least in part, by an MIF-mediated mechanism in LPS-stimulated macrophages but not in fMLP-stimulated PMN cells. The present study provides the first evidence supporting MIF as a mechanism for inflammation and immune modulation by HTS in LPS-stimulated macrophages. MIF appears to be a promising candidate for the treatment of sepsis in traumatic conditions.

## References

[1] Tsiotou AG, Sakorafas GH, Anagnostopoulos G, Bramis J (2005). Septic shock; current pathogenetic concepts from a clinical perspective. Med Sci Monit.

[2] Keel M, Trentz O (2005). Pathophysiology of polytrauma. Injury.

[3] Faist E, Baue AE, Dittmer H, Heberer G (1983). Multiple organ failure in polytrauma patients. J Trauma.

[4] Moore FA, Moore EE (1995). Evolving concepts in the pathogenesis of postinjury multiple organ failure. Surg Clin North Am.

[5] Coimbra R, Hoyt DB, Junger WG, Angle N, Wolf P, Loomis WH, Evers MF (1997). Hypertonic saline resuscitation decreases susceptibility to sepsis after hemorrhagic shock. J Trauma.

[6] Junger WG, Coimbra R, Liu FC, Herdon-Remelius C, Junger W, Junger H, Loomis WH, Hoyt DB, Altman A (1997). Hypertonic saline resuscitation: a tool to modulate immune function in trauma patients?. Shock.

[7] Angle N, Hoyt DB, Coimbra R, Liu F, Herdon-Remelius C, Loomis W, Junger WG (1998). Hypertonic saline resuscitation diminishes lung injury by suppressing neutrophil activation after hemorrhagic shock. Shock.

[8] Staudenmayer KL, Maier RV, Jelacic S, Bulger EM (2005). Hypertonic saline modulates innate immunity in a model of systemic inflammation. Shock.

[9] Fan J, Marshall JC, Jimenez M, Shek PN, Zagorski J, Rotstein OD (1998). Hemorrhagic shock primes for increased expression of cytokine-induced neutrophil chemoattractant in the lung: role in pulmonary inflammation following lipopolysaccharide. J Immunol.

[10] Botha AJ, Moore FA, Moore EE, Fontes B, Banerjee A, Peterson VM (1995). Postinjury neutrophil priming and activation states: therapeutic challenges. Shock.

[11] Kuchkina NV, Orlov SN, Pokudin NI, Chuchalin AG (1993). Volume-dependent regulation of the respiratory burst of activated human neutrophils. Experientia.

[12] Hampton MB, Chambers ST, Vissers MC, Winterbourn CC (1994). Bacterial killing by neutrophils in hypertonic environments. J Infect Dis.

[13] Davreux CJ, Soric I, Nathens AB, Watson RW, McGilvray ID, Suntres ZE, Shek PN, Rotstein OD (1997). N-acetyl cysteine attenuates acute lung injury in the rat. Shock.

[14] Powers KA, Zurawska J, Szaszi K, Khadaroo RG, Kapus A, Rotstein OD (2005). Hypertonic resuscitation of hemorrhagic shock prevents alveolar macrophage activation by preventing systemic oxidative stress due to gut ischemia/reperfusion. Surgery.

[15] Bernhagen J, Calandra T, Bucala R (1998). Regulation of the immune response by macrophage migration inhibitory factor: biological and structural features. J Mol Med (Berl).

[16] Daigneault M, Preston JA, Mariott HM, Whyte MK, Dockrell DH (2010). The identification of markers of macrophage differentiation in PMA-stimulated THP-1 cells and monocyte-derived macrophages. PLoS One.

[17] Schaeffer V, Arbabi S, Garacia IA, Knoll ML, Cuschieri J, Bulger EM, Maier RV (2011). Role of the mTOR Pathway in LPS-Activated Monocytes: influence of hypertonic saline. J Surg Res.

[18] Himmelseher S (2007). Hypertonic saline solutions for treatment of intracranial hypertension. Curr Opin Anaesthesiol.

[19] Bulger EM, Jurkovich GJ, Nathens AB (2008). Hypertonic resuscitation of hypovolemic shock after blunt trauma: a randomized controlled trial. Arch Surg.

[20] Poli-de-Figueiredo LF, Cruz RJ, Sannomiya P, Rocha-E-Silva M (2006). Mechanisms of action of hypertonic saline resuscitation in severe sepsis and septic shock. Endocr Metab Immune Disord Drug Targets.

[21] Deitch EA, Shi HP, Feketeova E (2003). Hypertonic saline resuscitation limits neutrophil activation after trauma-hemorrhagic shock. Shock.

[22] Chen Y, Hashiguchi N, Yip L, Junger WG (2006). Hypertonic saline enhances neutrophil elastase release through activation of P2 and A3 receptors. Am J Physiol Cell Physiol.

[23] Choi SH, Lee SW, Hong YS (2006). Selective inhibition of polymorphonuclear neutrophils by resuscitative concentration of hypertonic saline. Emerg Med J.

[24] Hashiguchi N, Lum L, Romeril E (2007). Hypertonic saline resuscitation: efficacy may require early treatment in severely injured patients. J Trauma.

[25] Hatanaka E, Shimomi FM, Curi R, Campa A (2007). Sodium chloride inhibits cytokine production by lipopolysaccharide-stimulated human neutrophils and mononuclear cells. Shock.

[26] Bloom BR, Bennett B (1966). Mechanism of a reaction in vitro associated with delayed-type hypersensitivity. Science.

[27] David JR (1966). Delayed hypersensitivity in vitro: its mediation by cell-free substances formed by lymphoid cell-antigen interaction. Proc Natl Acad Sci USA.

[28] Nathan CF, Karnovsky ML, David JR (1971). Alterations of macrophage functions by mediators from lymphocytes. J Exp Med.

[29] Nathan CF, Remold HG, David JR (1973). Characterization of a lymphocyte factor which alters macrophage functions. J Exp Med.

[30] Vulcano M, Alves Rosa MF, Minnucci FS, Cherñavsky AC, Isturiz MA (1998). N-formyl-methionyl-leucyl-phenylalanine (fMLP) inhibits tumour necrosis factor-alpha (TNF-alpha) production on lipopolysaccharide (LPS)-stimulated human neutrophils. Clin Exp Immunol.

[31] Stewart I, Schluter PJ, Shaw GR (2006). Cyanobacterial lipopolysaccharides and human health - a review. Environ Health.

[32] Snyderman R (1984). Regulation of Leukocyte Function.

[33] Yoon YH, Choi SH, Hong YS, Lee SW, Moon SW, Cho HJ (2011). Effect of hypertonic saline and macrophage migration inhibitory factor in restoration of T cell dysfunction. J Korean Surg Soc.

[34] Schmidt-Supprian M, Murphy C, While B, Lawler M, Kapurniotu A, Voelter W (2000). Activated protein C inhibits tumor necrosis factor and macrophage migration inhibitory factor production in monocytes. Eur Cytokine Netw.

